# Identification and Assessment of Octreotide Acylation in Polyester Microspheres by LC–MS/MS

**DOI:** 10.1007/s11095-015-1685-3

**Published:** 2015-04-02

**Authors:** Mehrnoosh Shirangi, Wim E. Hennink, Govert W. Somsen, Cornelus F. van Nostrum

**Affiliations:** 1 Department of Pharmaceutics, Utrecht Institute for Pharmaceutical Sciences, Utrecht University, Utrecht, The Netherlands; 2 AIMMS Division of Biomolecular Analysis, VU University Amsterdam, Amsterdam, The Netherlands; 3 Department of Drug and Food Control, Faculty of Pharmacy, Tehran University of Medical Science, Tehran, Iran

**Keywords:** acylation, aliphatic polyester, LC–MS/MS, octreotide

## Abstract

**Purpose:**

Polyesters with hydrophilic domains, *i.e.*, poly(d,l-lactic-*co*-glycolic-*co*-hydroxymethyl glycolic acid) (PLGHMGA) and a multiblock copolymer of poly(ε-caprolactone)-PEG-poly(ε-caprolactone) and poly(l-lactide) ((PC-PEG-PC)-(PL)) are expected to cause less acylation of encapsulated peptides than fully hydrophobic matrices. Our purpose is to assess the extent and sites of acylation of octreotide loaded in microspheres using tandem mass spectrometry analysis.

**Methods:**

Octreotide loaded microspheres were prepared by a double emulsion solvent evaporation technique. Release profiles of octreotide from hydrophilic microspheres were compared with that of PLGA microspheres. To scrutinize the structural information and localize the actual modification site(s) of octreotide, liquid chromatography ion-trap mass spectrometry (LC-ITMS) was performed on the acylated adducts.

**Results:**

Hydrophilic microspheres showed less acylated adducts in comparison with PLGA microspheres. LC-MS/MS showed that besides the N-terminus and primary amine of lysine, the primary hydroxyl of the end group of octreotide was also subjected to acylation. Nucleophilic attack of the peptide can also occur to the carbamate bond presented in (PC-PEG-PC)-(PL) since 1,4-butanediisocyanate was used as the chain extender.

**Conclusions:**

Hydrophilic polyesters are promising systems for controlled release of peptide because substantially less acylation occurs in microspheres based on these polymers. LC-ITMS provided detailed structural information of octreotide modifications *via* mass analysis of ion fragments.

**Electronic supplementary material:**

The online version of this article (doi:10.1007/s11095-015-1685-3) contains supplementary material, which is available to authorized users.

## Introduction

In recent years, there has been an increasing interest in formulations for therapeutic peptides and proteins based on aliphatic polyesters to achieve targeted and/or sustained release of these therapeutics (1–3). Particularly microspheres of poly(d,l-lactic-*co*-glycolic acid) (PLGA) have been extensively used for prolonged release of bioactive peptides and proteins (4–6). However, one of the major obstacles in formulation of therapeutic peptides/proteins with PLGA is the modification of the actives as a result of acylation with lactic and glycolic units. Nucleophilic groups, particularly the N-terminus and primary amine groups of lysine residues, attack the electrophilic carbonyls of the ester groups of the PLGA backbone, which results in covalent addition of glycolyl or lactyl groups on the released peptide (7,8). It was found that the low pH that is generated inside degrading PLGA microspheres catalyzes these acylation reactions (9). Importantly, acylation can result in unwanted change of activity, immunogenicity and toxicity due to the structural changes of peptides/proteins (10,11) and should therefore be avoided.

Octreotide is a synthetic octapeptide analogue of somatostatine which is clinically used for the treatment of acromegaly as well as certain endocrine tumors (12). Octreotide has poor pharmacokinetics (half-life of 100 min in humans) and therefore a sustained formulation based on PLGA microspheres has been developed which is presently used in the clinic (13). Octreotide has a free N-terminus and a lysine amine group and has therefore been studied in depth regarding acylation reactions that occur in matrices of PLGA and related aliphatic polyesters (14–20). For instance, Murty *et al.* (21) studied the acylation of octreotide acetate, formulated in microspheres of PLGA of varying molecular weight and comonomer composition. They found that due to the steric hindrance of the α-methyl groups of lactic acid units as compared to glycolic acid units, PLGA polymers with higher lactic acid content were less amenable to formation of acylation adducts as compared to PLGA with higher glycolic acid content. They also showed that microspheres prepared from PLGA 50:50 (9 kDa) released 54% of loaded octreotide within 50 days and, importantly, 66% of the released peptide was acylated. Sophocleous *et al.* studied the nature of peptide interaction with PLGA to get insight how peptide or PLGA properties effect sorption and acylation. They suggested that peptide sorption to PLGA is the first step to peptide acylation. They prevented the peptide sorption to PLGA by adding divalent cationic salt which indeed resulted to attenuation of acylation (22). Ghassemi *et al.* compared the acylation of octreotide that occurs in poly(lactic-*co*-hydroxymethyl glycolic acid) (PLHMGA) microspheres (23,24), with the commercial octreotide microsphere formulation (Sandostatin LAR®, which is based on PLGA-glucose star polymer). They found that less acylated octreotide adducts were formed in PLHMGA microspheres than in Sandostatin LAR® most likely due to the lack of glycolic acid units and lower extent of acidification during degradation (9,18). In the studies mentioned above, it was shown that the extent of acylation depends on the type of polymer used. Further, no detailed information has been reported at which site acylation preferentially occurs (N-terminus or lys). Therefore, in the present study we further explored acylation and prevention of acylation using more hydrophilic matrices than PLGA and also assessed the sites of acylation using tandem mass spectrometry analysis. To this end, octreotide was encapsulated in microspheres based on PLGHMGA (poly(lactic-*co*-glycolic-*co*-hydroxymethyl glycolic acid)), *i.e.*, copolymers containing hydrophilic HMGA units but also containing glycolic acid units (25), as well as those based on a multiblock copolymer of poly(ε-caprolactone)- PEG-poly (ε-caprolactone) with poly(l-lactide)). LC–MS/MS analysis was applied to identify the extent and sites of acylation by using ion-trap mass spectrometry (ITMS) employing electrospray ionization (ESI).

## Materials and Methods

### Chemicals

**Fig. 1:**
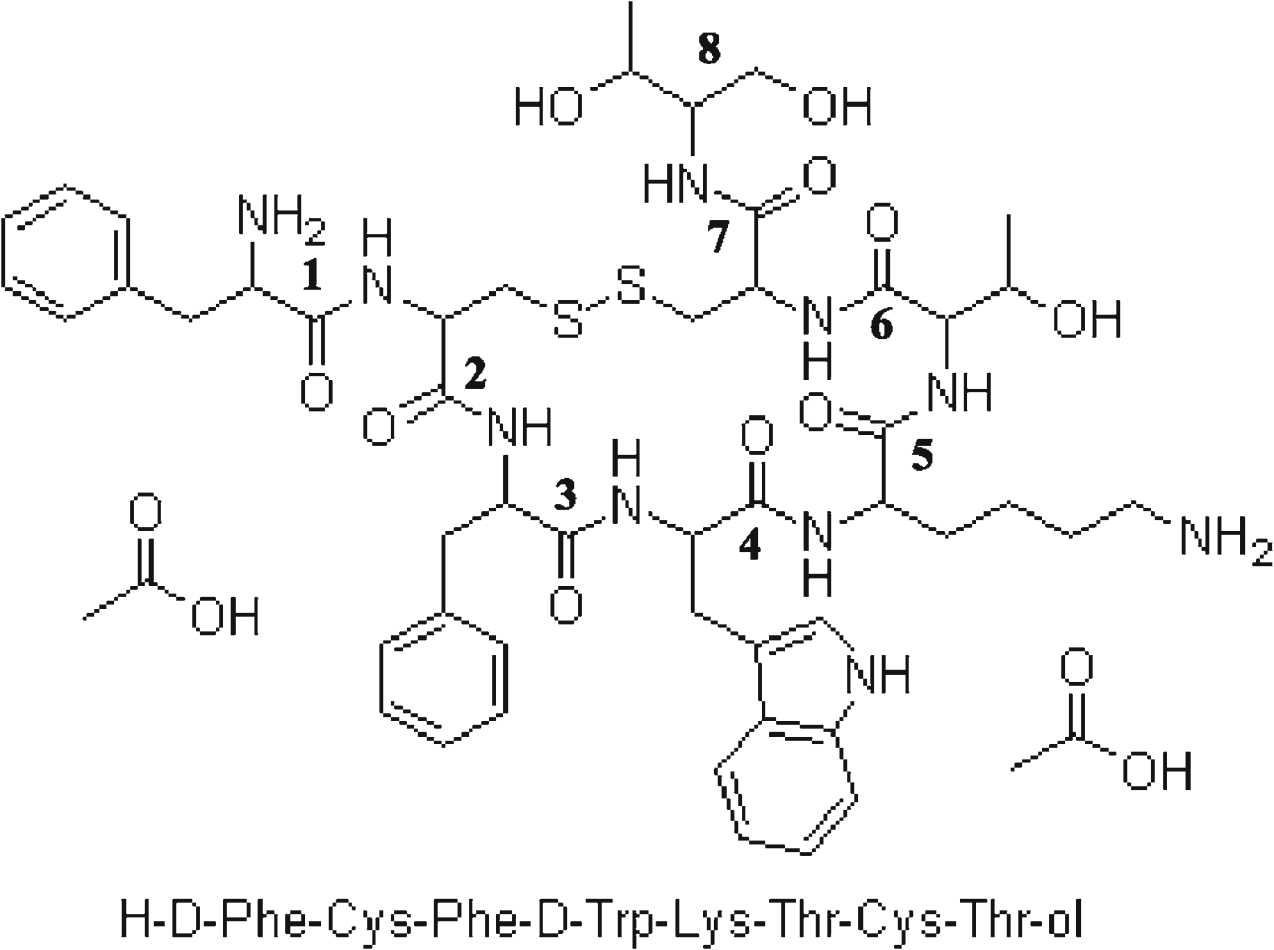
Structure of octreotide acetate.

Octreotide acetate (H_2_N-D-Phe-Cys-Phe-D-Trp-Lys-Thr-Cys-Thr-ol, MW = 1018.8 Da; Fig. 1) was obtained from Feldan-bio (Quebec, Canada). Polyvinyl alcohol (PVA; MW 30,000–70,000; 88% hydrolyzed) was from Sigma-Aldrich, Inc., USA. Disodium hydrogen phosphate dihydrate (Na_2_HPO_4_^.^2H_2_O) and sodium dihydrogen phosphate monohydrate (NaH_2_PO_4_^.^H_2_O) were obtained from Merck. Sodium azide (NaN_3_, Bio Ultra, ≥99.5%) was purchased from Sigma (Germany). HPLC and MS grade acetonitrile (ACN), peptide grade dichloromethane (DCM) and tetrahydrofurane (THF) were purchased from Biosolve (The Netherlands). Formic acid was purchased from Sigma-Aldrich Co (Zwijndrecht, the Netherlands). Dithiothreitol was from Sigma-Aldrich Co (Canada).

**Fig. 2:**
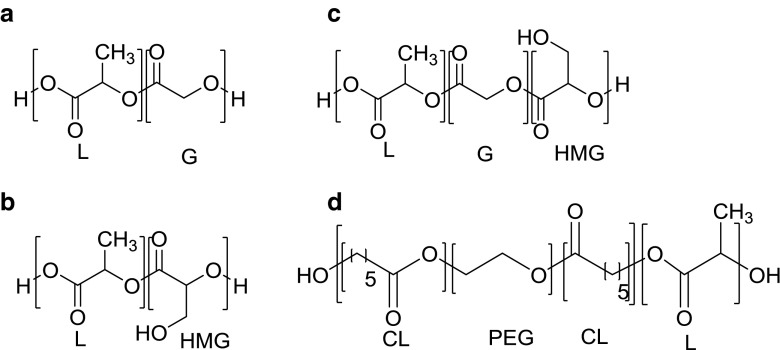
Structural formulas of the polymers used in this study: (**a**) Poly(d,l-lactic-*co*-glycolic acid) (PLGA 50/50), (**b**) Poly(d,l-lactic-*co*-hydroxymethyl glycolic acid) (PLHMGA 82/18), (**c**) poly(d,l-lactic-*co*-glycolic-*co*-hydroxymethyl glycolic acid) (PLGHMGA 64/18/18), (**d**) poly((ε-caprolactone-*b*-PEG-*b*-ε-caprolactone)-*b*-l-lactide)) (PC-PEG-PC)-(PL).

PLGA (acid terminated 5004A with d,l-lactide/glycolide molar ratio 50:50, IV = 0.4 dl/g) was obtained from Purac, The Netherlands. Poly(lactic-*co*-hydroxymethyl glycolic acid) (PLHMGA) (82/18 lactic/hydroxymethyl glycolic acid ratio (L/HMG) and *M*_w_ of 22.5 kg/mol) and poly(lactic-*co*-glycolic-*co*-hydroxymethyl glycolic acid) (PLGHMGA) (64/18/18 lactic/glycolic/hydroxymethyl glycolic acid (L/G/HMG) ratio, *M*_w_ of 44 kg/mol) were synthesized as described by Leemhuis *et al.* (23,24). The Polymers are further referred to as PLGA, PLHMGA and PLGHMGA, respectively. The company Innocore (The Netherlands) kindly provided 20CP10C20-LL40 (IV = 0,67 dl/g), which is a multiblock copolymer that was prepared by chain extension of a ABA-triblock copolymer of poly(ε-caprolactone) and 1000 Da PEG with a poly(l-lactide) block with a molecular weight of 4000, using 1,4-butanediisocyanate as a chain extender (26,27). This copolymer is further referred to as (PC-PEG-PC)-(PL). The polymer structures are shown in Fig. 2.

### Microspheres Preparation

Octreotide loaded microspheres were prepared by a double emulsion (W/O/W) solvent evaporation technique (18). Briefly, 50 μl of an octreotide solution in milliQ water (200 mg/ml) was emulsified in 500 μl of a dichloromethane solution of polymer (220 mg, 25% *w*/*w*) by using an IKA homogenizer (IKA Labortechnik Staufen, Germany) for 30 s at the highest speed (30,000 rpm) to get the primary emulsion. Next, 500 μl of a PVA solution (1% *w*/*w* in 30 mM sodium phosphate buffer, pH 7.4) was added, and the mixture was vortexed for 30 s at 30,000 rpm. The w/o/w emulsion was subsequently transferred into an external aqueous solution (5 ml) containing PVA 0.5% (*w*/*w*) in 30 mM phosphate buffer pH 7.4 while stirring. Continuous stirring at room temperature for 2 h resulted in extraction/evaporation of DCM. Finally, hardened microspheres were collected by centrifugation (Laboratory centrifuge, 4 K 15 Germany) at 3000 g for 3 min, subsequently washed 3 times with 50 ml RO water and freeze dried at −50°C and at 0.5 mbar in a Chris Alpha 1–2 freeze-dryer (Osterode am Harz, Germany) for 16 h. The dried microspheres were stored at −25°C.

### Characterization of the Microspheres

The Microspheres’ size and size distribution were analyzed by a laser blocking technology (Accusizer 780, Optical particle sizer, Santa Barbara, California, USA) after dispersing the freeze dried microspheres in water. The morphology of the freeze dried microspheres was analyzed by scanning electron microscopy using a Phenom^™^ SEM (FEI Company, the Netherlands). The samples were mounted onto a 12 mm diameter aluminum specimen stub (Agar Scientific Ltd., England) using double-sided adhesive tape and were coated with 6 nm platinum prior to analysis. The octreotide loading was determined by dissolving about 10 mg of microspheres in 2 ml of THF with gentle shaking. Thereafter, 2 ml of solution of 0.2% *w*/*v* glacial acetic acid, 0.2% *w*/*v* sodium acetate and 0.7% *w*/*v* sodium chloride in water was added to precipitate the polymer. Next, the mixture was incubated at room temperature for 20 min, and the precipitated polymer was spun down by centrifugation at 5000 g for 2 min. The octreotide content in the supernatant was measured by ultra performance liquid chromatography (Waters ACQUITY UPLC®) using an ACQUITY BEH 300 C18 column (1.7 μm, 2.1 × 50 mm). A gradient elution method was used with a mobile phase A (95% H_2_O, 5% ACN + 0.1% TFA) and a mobile phase B (100% ACN + 0.1% TFA). The eluent linearly changed from 100 to 70% B in 5 min with a flow rate of 0.25 ml/min. Octreotide standards (5–100 μg/ml, 7 μl injection volume) were used for calibration, and detection was done both at UV 210 nm and using fluorescence with setting excitation at 280 nm and emission a 330 nm. Loading efficiency (LE) of the peptide in the microspheres is reported as the encapsulated peptide divided by the total amount of peptide used for encapsulation. Loading capacity (LC) is defined as the encapsulated amount of octreotide divided by dry weight of the microspheres.

#### ***In Vitro*** Release Studies

Octreotide release from different microspheres was studied in PBS (0.033 M NaH_2_PO_4_, 0.066 M Na_2_HPO_4_, 0.056 M NaCl and 0.05% (*w*/*w*) NaN_3,_ pH 7.4). About 30 mg of microspheres (accurately weighted) were suspended into 1.5 ml of PBS buffer in eppendorf tubes and incubated at 37°C under mild agitation using a circular mixer (ASSISTANT RM 5). At the different time points, the dispersion was centrifuged (3000 g, 3 min), and 1 ml of the supernatant was replaced with 1 ml of fresh buffer. The microspheres were resuspended by gentle shaking, and the dispersion was incubated at 37°C. The released samples were kept in −20°C until measurement by UPLC and mass analysis.

### LC–MS/MS Analysis

LC–MS experiments were performed using a Shimadzu 10A HPLC system (Kyoto, Japan) coupled to an Agilent Technologies 6300 Series LC/MSD ion-trap mass spectrometer (Santa Clara, CA, USA). A HPLC column (150 × 4.6 mm) packed with 3.5 μm XBridge^™^ BEH130 C18 material was used at ambient temperature.

A gradient method was used with a mobile phase A (95% H_2_O, 5% ACN + 0.1% formic acid) and a mobile phase B (100% ACN + 0.1% formic acid). The eluent linearly changed from 100% A to 100% B in 20 min with a flow rate of 0.5 ml/min. The injection volume was 10 μl. Electrospray ionization (ESI) was performed in the positive ion mode using an Agilent Technologies ion source and interface. The MS settings were: capillary voltage,2 kV; nebulizer pressure, 60 psi; dry gas flow, 11 L/min; dry gas temperature, 350°C; scan range, *m/z* 50–1500. MS/MS experiments were performed using an isolation width of 2 Da and a fragmentation amplitude of 1.0 V.

Since the disulfide bond between the two cysteine residues in octreotide hinders peptide fragmentation in the mass spectrometer, 500 μl of the released octreotide samples were incubated with 50 μl of dithiothreitol (DDT) 10 mg/ml in water at 37°C for 4 h before MS/MS analysis (28).

## Results and Discussions

### Preparation and Characterization of Octreotide-Loaded Microspheres

**Fig. 3:**
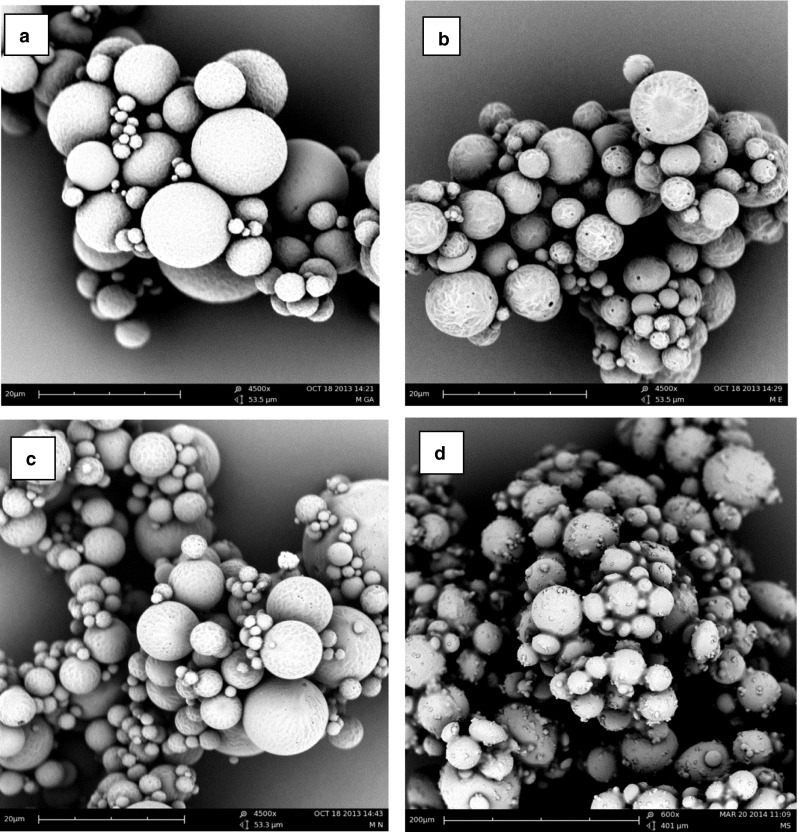
SEM pictures of octreotide loaded microspheres based on (**a**) PLGA, (**b**) PLHMGA, (**c**) PLGHMGA, (**d**) (PC-PEG-PC)-(PL).

Octreotide-loaded microspheres of the different aliphatic polymers of different composition and hydrophilicity (structures shown in Fig. 2) were prepared using a common double emulsion/solvent evaporation method. The characteristics of the microspheres are summarized in Table I. The microspheres have the average size ranging from 20 to 53 μm and the loading capacity varies between 2.1 and 2.8%. SEM pictures are shown in Fig. 3, and demonstrate that particles are spherical and have some pores (formulation B and D) or are non-porous (formulation A and C).

**Table I:** Characteristics of Octreotide-Loaded Microspheres (*n* = 3)

Polymer	Volume weight diameter (μm)	LE^a^ (%)	LC^b^ (%)
PLGA	20.5 ± 3.0	54.1 ± 5.1	2.5 ± 0.2
PLGHMGA	23.7 ± 2.5	58.1 ± 6.1	2.7 ± 0.3
PLHMGA	24.8 ± 3.3	62.9 ± 7.5	2.8 ± 0.3
(PC-PEG-PC)-(PL)	53.6 ± 3.5	45.9 ± 4.2	2.1 ± 0.2

^a^ *LE* loading efficiency

^b^ *LC*, loading capacity

#### ***In Vitro*** Release of Octreotide-Loaded Microspheres

**Fig. 4:**
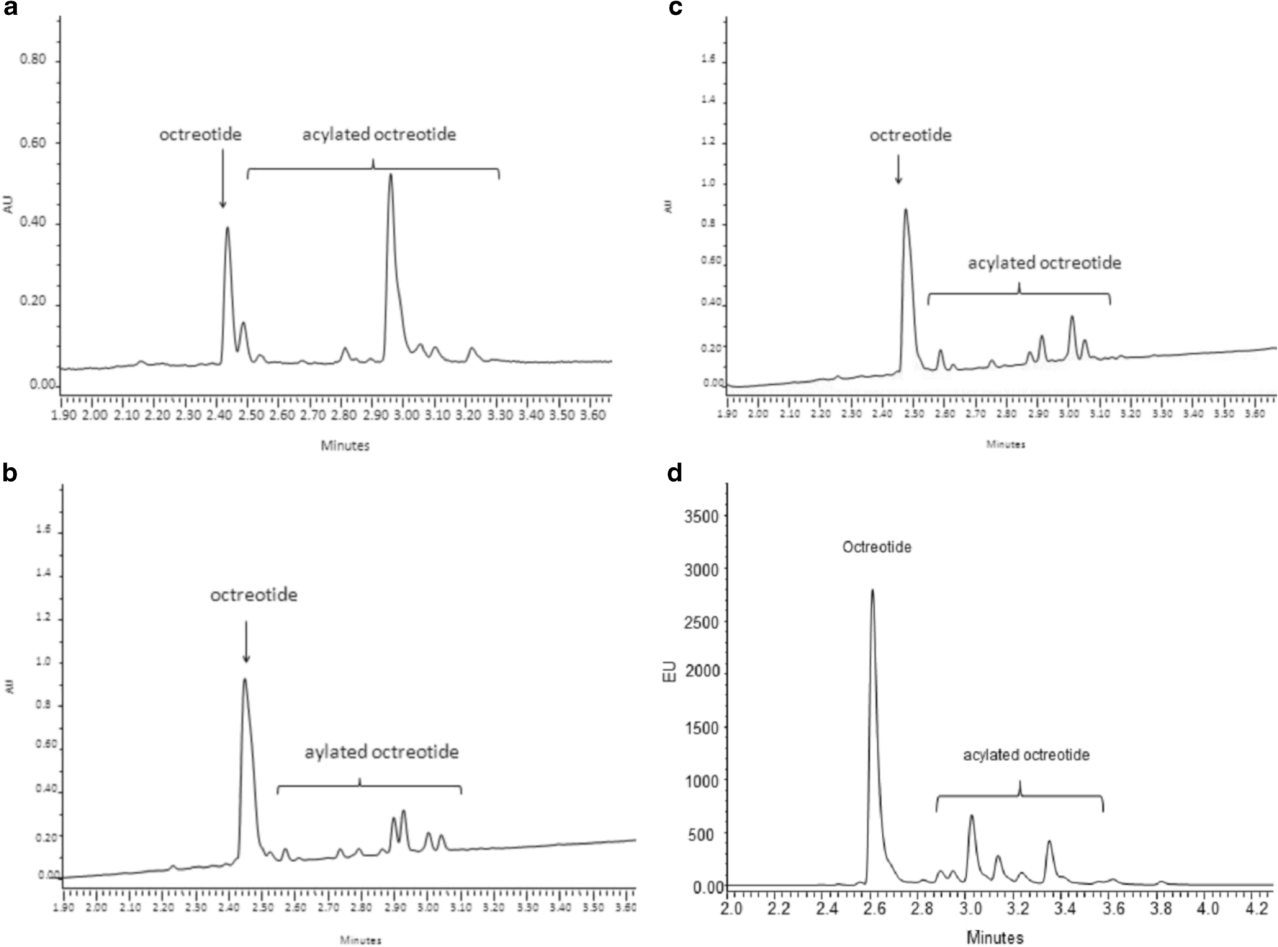
UPLC of octreotide released after 45 days in PBS pH 7.4 at 37°C from (**a**) PLGA, (**b**) PLGHMGA, (**c**) PLHMGA and (**d**) (PC-PEG-PC)-(PL) (the latter after 28 days).

UPLC-UV chromatograms of octreotide released after 45 days from PLGA, PLGHMGA and PLHMGA, respectively, and from (PC-PEG-PC)-(PL) after 28 days, are shown in Fig. 4. The main peak eluting after approx. 2.5 min corresponds to native octreotide, while the extra peaks with longer retention times are originating from acylated octreotide adducts (see LC–MS analysis). It is remarkable that the extent of acylated adduct is significantly more pronounced for octreotide released from PLGA microspheres than that released from the other microspheres. It should be noted that only native octreotide was detected when the microspheres were dissolved immediately after preparation, demonstrating that the manufacturing process did not cause the acylation of the peptide. In line herewith, it has been observed that acylation of peptides occurs during the *in vitro* release from PLGA microspheres when the polymer starts to degrade (14). Peptide acylation is catalyzed by the low pH that is generated inside degrading PLGA microspheres due to the accumulation of acid degradation products, *i.e.*, glycolic and lactic acid and soluble oligomers thereof (9,14,29,30).

**Fig. 5:**
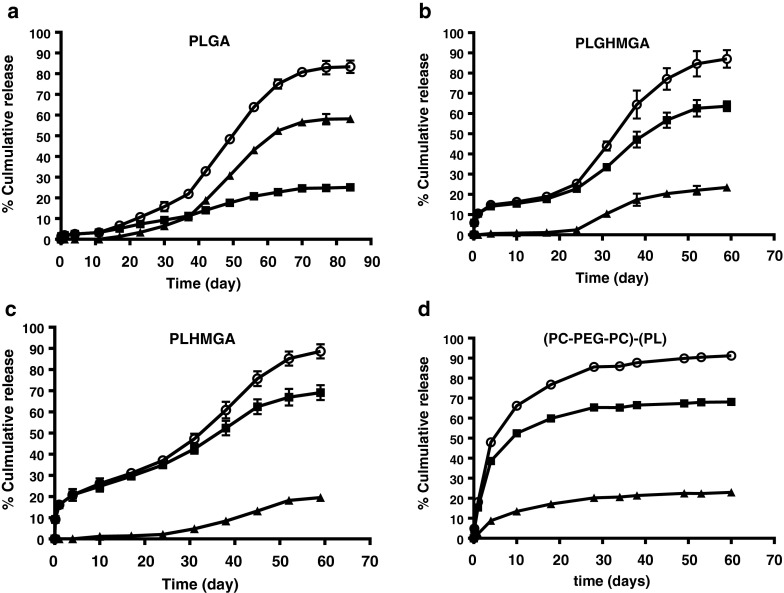
*In vitro* release of octreotide from (**a**) PLGA, (**b**) PLGHMGA, (**c**) PLHMGA and (**d**) (PC-PEG-PC)-(PL) microspheres in PBS pH 7.4: native octreotide (*squares*), acylated octreotide (*triangles*) and total octreotide (sum of native and acylated adducts, *circles*).

Figure 5 shows the cumulative release of octreotide from the different microspheres in PBS pH 7.4 at 37°C. These graphs show that acylation of octreotide is significantly higher when released from PLGA microspheres than of the others. Peptide release from PLGA microspheres started after 15–20 days without burst release and continued for the next 70 days until a plateau at 80% of the loaded amount was reached. UPLC analysis showed that only 31% of the released peptide was in its native form, while 69% was acylated (assuming the same UV-absorbance response for native and acetylated product). PLGHMGA microspheres showed after a burst release of 10% consisting of only native peptide, a phase of low release of about 25 days (~10% of the loading was released). Faster release of octreotide (both native and acylated) started at day 25 reaching 90% of the loading at day 60. UPLC analysis showed that 72% of the released octreotide was in its native form while only 28% was acylated. For PLHMGA microspheres, in line with the result of PLGHMGA, initially only native octreotide was released, and both native and acylated peptide started to be released after day 25. Finally, around 78% of the released peptide was native octreotide, whereas 22% was acylated adducts. For (PC-PEG-PC)-(PL) microspheres, the release was faster than from the other formulations and it seems that the release is governed by diffusion rather than polymer degradation (26). This is understandable because PEG increases the hydrophilicity of the polymer matrix which in turn results in water absorption allowing diffusion of peptide through the (channels or pores of) hydrated particles. The (PC-PEG-PC)-(PL) microspheres particles released the peptide in a continuous manner for 30 days and >90% of the loading was released at that time point. UPLC analysis showed that 75% of the released octreotide was in its native form while 25% was acylated. Figure 4d also shows that both native and acylated peptides were released from the start of the experiment. Although the presence of polymer degradation products can catalyze peptide acylation, the role of water should be considered as well. Liang *et al.* showed that a bell shape relation exists between the water content and the extent of acylation of exenatide (a polypeptide drug with a molecular weight of 4200 Da, which is clinically used as an adjunct for glycemic control in type 2 diabetes) when drug-loaded PLGA microspheres were incubated at different relative humidity. They showed that acylation kinetics depends on the water content of the microspheres. At low water contents, water acts as plasticizer resulting in more acylation of the unfolded peptide (unfolding occurs due to the hydrophobic polymer matrix). According to the authors, at higher water contents, bulk water present in the matrix will cause conformation recovery of the peptide resulting in a state in which it is less susceptible for acylation reaction (31). The presence of PEG in (PC-PEG-PC)-(PL) microspheres will result in rapid hydration of the particles during the initial stage of the incubation in buffer and facilitate acylation of octreotide. This explains that already at early time points of release acylated octreotide adducts were detected (Fig. 5d). However at later time points the ratio between native and acylated octreotide did not change suggesting that acylation occurs only during the initial stages likely because in later stages the water content of the micrsopheres became so high that acylation is prevented. Further the high water content facilitates release of the polymer degradation products.

The observation that acylation adduct formation of octreotide in PLGA (50/50) was substantially higher than in the other polymers can be explained by its high glycolic acid content and by the low water content that favors interactions between the peptide and the polymer matrix. Octreotide released form either PLGHMGA or PLHMGA microspheres was substantially less acylated than the peptide released from PLGA. Most likely, the hydroxyl pendent groups of PLHMGA and PLGHMGA increase the water absorption of the particles and facilitates the release of formed acid degradation products (18). Further, octreotide released from PLGHMGA microspheres (containing glycolic acid as monomer) was slightly more acylated than the peptide released from PLHMGA microspheres lacking glycolic acid monomers (28 and 22% respectively). Indeed, nucleophile attack is less hindered with glycolic ester units as compared to hydroxymethyl glycolic ester and lactic ester units (21).

The particle size studied in this paper is slightly smaller than most depots. Although some papers (*e.g.*, Dunne *et al.* (32)) show that the polymer degradation rate (and release profile) depends on the particle size, it is difficult to predict the rate of acylation with particle size. One effect could be that peptides have a shorter pathway to diffuse through the pores in small particles, and the diffusion of degraded polymers such as monomer and oligomer is also easier, from which it might be anticipated that the peptide is in less contact with polymers and less acylation occurs. On the other hand, if the accumulation of acidic oligomer inside the bigger particles cause autocatalytic degradation of polymer and faster release of the peptide may cause easier release and less acylation.

### LC–MS & LC–MS/MS Analysis

LC–MS analysis confirmed that the extra peaks in the chromatograms (Fig. 4) indeed represent acylated adducts. The assignments of observed masses of products released from PLGA, PLHMGA and PLGHMGA microspheres are summarized in Table II.

**Table II:** Acylated Octreotide Adducts Observed by LC-ESI-MS in Release Samples of PLGA, PLHMGA and PLGHMGA Microspheres

Observed [M + H]^1+^ m/z	Assigned structure of peptides			
	Δm	PLGA	PLHMGA	PLGHMGA
1021^a^	0	Octreotide	Octreotide	Octreotide
1079	+58	Octreotide-GA	N.D.	Octreotide-GA
1093	+72	Octreotide-LA	Octreotide-LA	Octreotide-LA
1109	+88	N.D.	Octreotide-HMGA	Octreotide-HMGA
1137	+116	Octreotide-GA-GA	N.D	Octreotide-GA-GA
1151	+130	Octreotide-GA-LA	N.D	Octreotide-GA-LA
1167	+146	N.D	N.D	Octreotide-GA-HMGA

*N.D.* not detected, GA, LA and HMGA are glycolyl, lactyl and hydroxymethyl glycolyl adducts, respectively

^a^The m/z of octreotide is 1019, the increase with 2 Da is due to the reduction of disulfide bond by DDT

**Fig. 6:**
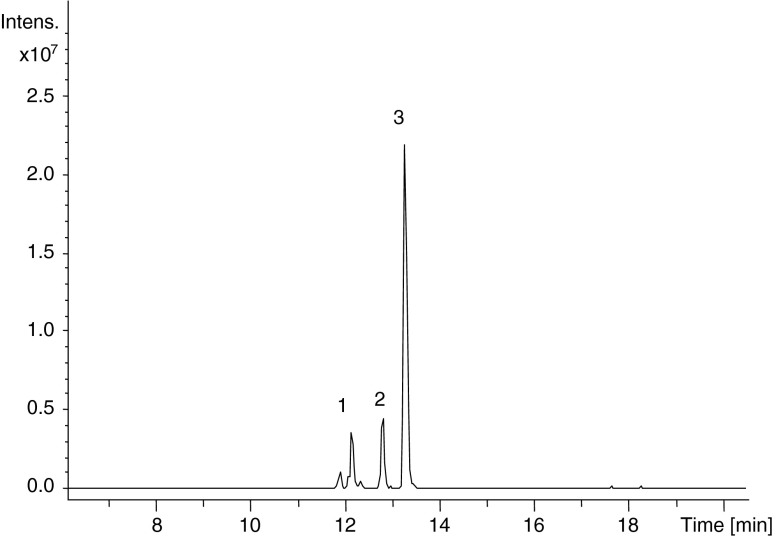
Extracted-ion chromatogram at m/z 1079 (= reduced octreotide, 1021 + 58) obtained by LC–MS of octreotide released from PLGHMGA after 45 days.

In order to exactly determine the extent and site of acylation LC–MS/MS was performed. With Bruker Compass DataAnalysis software, the m/z values of the analyte of interest were extracted from the entire data set for each chromatographic run. For example, the extracted ion chromatogram (EIC) at m/z 1079 which is ascribed to reduced octreotide + GA (1021 + 58) showed 3 main peaks with retention times of 12.1, 12.8 and 13.3 min (Fig. 6).

**Fig. 7:**
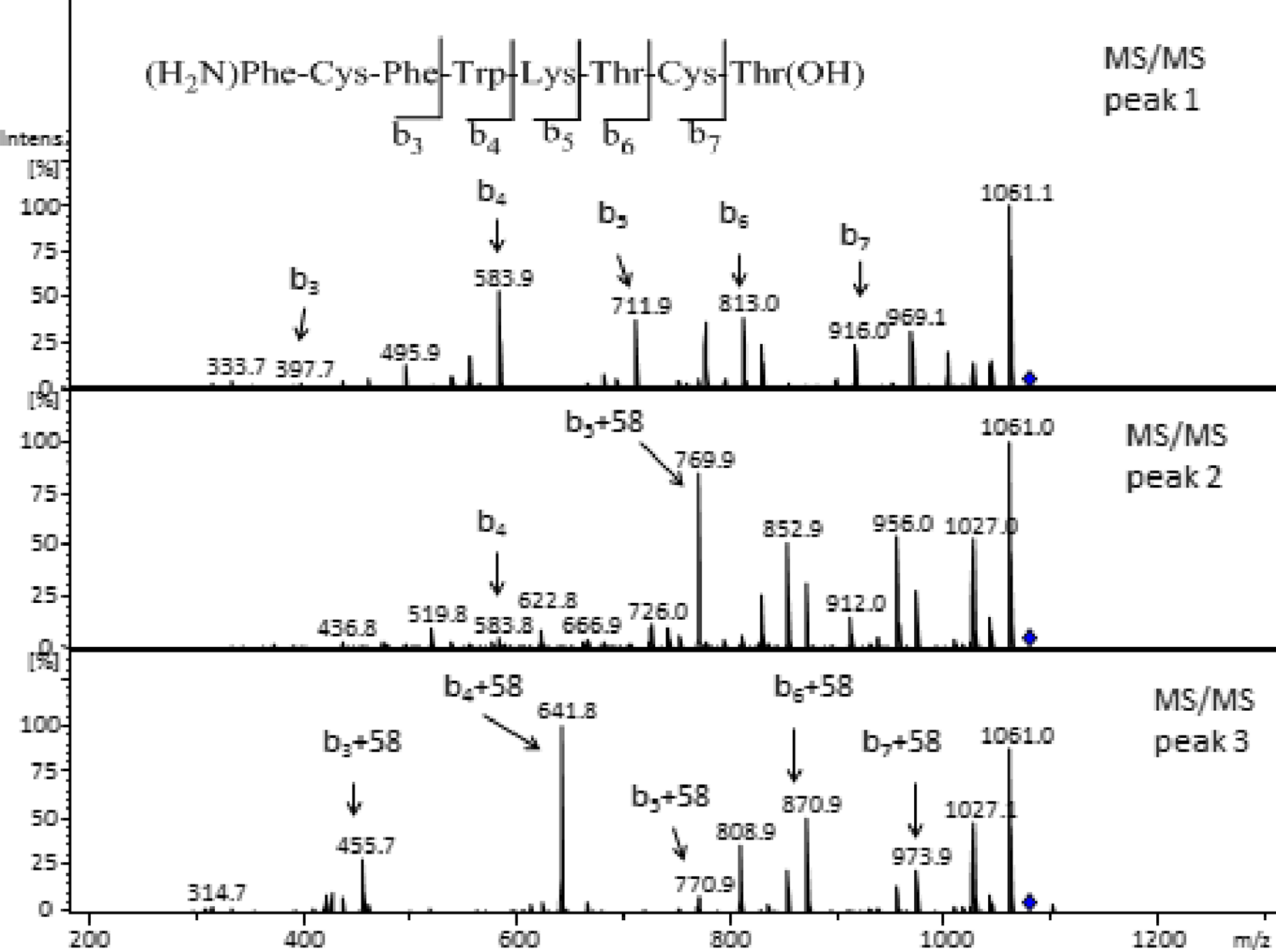
MS/MS spectra of observed peaks in Fig. 6 at m/z 1079.

**Fig. 8:**
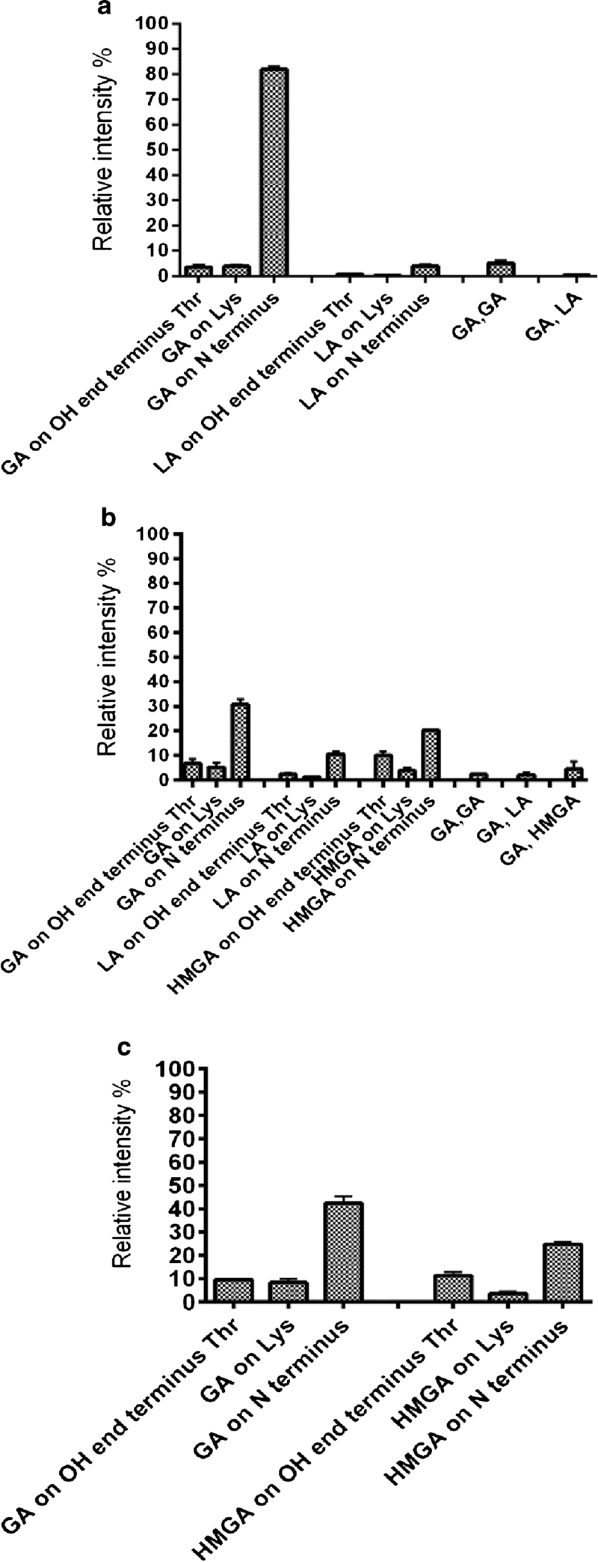
Relative intensity of released acylated adducts of octreotide after 45 days from microspheres of (**a**) PLGA (**b**) PLGHMGA (**c**) PLHMGA.

Figure 7 shows the LC–MS/MS analysis of the peaks observed in the EIC at m/z 1079. MS/MS revealed complete amino acid sequence information by a comprehensive series of b-ions which carry the N-terminus. The MS/MS of peak 1 (in Fig. 6) shows that all b ions up to b_7_ can be attributed to the sequence of the native peptide, indicating that acylation had occurred on the last amino acid (position 8, Fig. 1). The MS/MS results however cannot distinguish whether the primary or the secondary hydroxyl group of this position 8 group has been acylated. However, no acylation was observed on the secondary hydroxyl of threonine at position 6 and therefore it is concluded that the primary hydroxyl of position 8 is susceptible for acylation. In the second peak of the chromatogram in Fig. 6, the b_4_ ion was found unaffected while b_5_ incremented by 58 Da, indicating that lysine (position 5) has been acylated. In the last peak (number 3), all b-ions where shifted by +58 Da, demonstrating that the amine of the N-terminus was acylated. The ion at m/z 1109 of octreotide released from PLGHMGA microspheres corresponds with addition of one HMGA unit to the native peptide (Table II). The extracted-ion chromatogram of this ion also showed 3 peaks pointing to the same sites of modification as observed for the octreotide-GA adduct (FigureS1 & S2). Figure S3 shows four peaks for the extracted ion chromatogram at m/z 1093, which corresponds with addition of one lactic acid unit to octreotide. The MS/MS spectrum (Figure S4) of the first peak indicates the addition of the LA unit on the primary hydroxyl of the terminal amino acid, whereas the second peak is ascribed to addition on Lys, while peaks 3 & 4 have the exact same fragmentation pattern both indicating the addition of LA on the N-terminus. We presume that this is the result of the formation of diastereoisomers because the polymer contained both d- and l-lactic acid units. The same analysis was performed for all the observed ions that are mentioned in Table II for the peptide released from the other two microspheres (PLGA and PLHMGA);the distributions of acylation adducts are summarized in Fig. 8. One can conclude that the N-terminus of octreotide is the most susceptible site for acylation, with the acylation by glycolic acid (if present in the copolymer) being the most abundant.

Lucke *et al.* applied enzymatic cleavage of salmon calcitonin (sCT) released from PLGA microspheres in order to identify the amino acids that are potential sites for acylation. They treated sCT with Endoproteinase Glu-C which selectively cleaves the peptide bond on the carboxyl site of glutamate. It was concluded that besides the N-terminal amine group, lysine, tyrosine and serine could be acylated, although without specifying exactly which amino acid were susceptible for derivatization (14). For octreotide, up to now only lysine and N-terminal phenylalanine were reported in the literature as the sites of acylation. Murty *et al.* reported that at least 11 additional peaks after the native octreotide peak were detected in the chromatogram of the product that was released from PLGA 50/50 microspheres. They identified 9 out of 11 observed acylated compounds and mentioned adduct formation at either the N-terminus or lysine residue with a higher degree of modification of the N-terminus. They also suggested the presence of diglycolyl, trigycolyl or lactyl-glycolyl adducts on the single lysine to explain the large number of different species formed (21). However, no convincing evidence was presented to substantiate these claims. We have no evidence of dimer or trimer addition on lysine. De Jong *et al.* showed that at acidic pH the hydrolysis of lactic acid oligomers proceeds *via* fairly rapid chain-end scission (33). Therefore, possible dimeric or trimeric aducts on the peptide will easily be hydrolyzed to form single adducts. Indeed, we now clearly show by LC-ITMS that three different species for each type of single acylated product can be identified (*i.e.*, acylation on the N-terminus, Lys and Thr). Including the different species that can theoretically be formed with the two glycolyl and with the one glycolyl plus one lactyl unit observed at m/z = 1137 and 1151, respectively (see Table II), this would give rise to 15 different possible combinations.

**Fig. 9:**
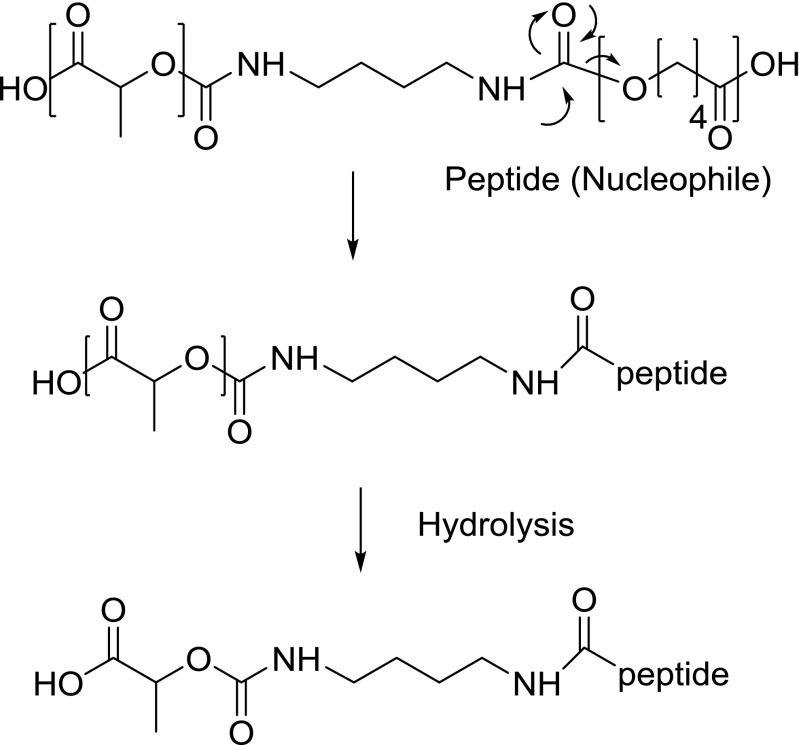
Proposed mechanism of reaction between peptide and carbamate group in (PC-PEG-PC)-(PL) multiblock copolymer.

For octreotide released from the (PC-PEG-PC)-(PL), two main peaks were observed in LC–MS with m/z values that were 72 and 212 Da higher than observed for native octreotide (Table III). The peak with m/z 1091 is ascribed to LA addition to octreotide. Since 1,4-butanediisocyanate has been used as the chain extender connecting the PC-PEG-PC with poly(lactic acid) to form the poly((ε-caprolactone-*b*-PEG-*b*-ε-caprolactone)-*b*-l-lactide)) multiblock copolymer, we propose that nucleophiles of the peptide can react with the carbonyl group of the carbamate bond (Fig. 9). Consequently, after full hydrolysis of the remaining ester groups, a butanediisocyanate bound by a carbamate bond to one lactic acid unit remains coupled to the peptide. Indeed, the molecular weight of this group is 212 Da and explains the presence of the m/z 1231 ion. It should be noted that for quantitative measurement of the acylated products of the peptide released from (PC-PEG-PC)-(PL) microspheres (Figs. 4d & 5d), fluorescence was used for detection in order to avoid the overestimation of acylated adducts at UV 210 because of the absorbance by the carbamate bond at this wavelength (34).

**Table III:** Acylated Octreotide Adducts Observed by LC-ESI-MS in Octreotide Released from (PC-PEG-PC)-(PL) Microspheres

Observed [M + H]^1+^ m/z	Δm	Assigned structure	% Relative intensity of released acylated adducts
1091	+72	Octreotide-LA	63.5 ± 2.5
1231	+212	Octreotide-butanediisocyanate-LA	36.5 ± 2.5

Table IV shows the monomer feed ratio for the synthesis (PC-PEG-PC)-(PL), as provided by the manufacturer. Interestingly, although the feed contained only 1.1 mole % of 1,4-butanediisocyanate (as compared with 75.9% of l-lactide), the relative intensities of the peaks in the LC–MS chromatogram were 63.5:36.5 for 1,4-butanediisocyanate-LA and LA, respectively. Likely, the carbonyl group of the carbamate group is more accessible for attack by the nucleophile of the peptide than the secondary ester groups of the PLLA blocks. Also the higher hydrophilicity of the carbamate group than the ester group might contribute to its higher reactivity. Importantly, no addition of ε-caprolactone of released octreotide was observed pointing to a low reactivity of the caprolactone ester groups. This is likely due to the hydrophobicity of the caprolactone blocks in combination with a low local solubility of the peptide in caprolactone rich domains.

**Table IV:** Monomer Feed Ratio for the Synthesis of (PC-PEG-PC)-(PL)

	wt.%	Mole %
1,4-butanediol	1.9	1.5
1,4-butanediisocyanate	2.2	1.1
ε-caprolactone	9.6	6
PEG1000	9.6	15.5
l-lactide	76.8	75.9

Interestingly, the extracted ion chromatogram of the octreotide adduct with one LA unit released from (PC-PEG-PC)-(PL) (see Figure S5), shows three peaks as opposed to the four peaks that we observed for the LA adducts of the other polymers (*vide supra*). This can be explained by the fact that for the synthesis of (PC-PEG-PC)-(PL) just l-lactide had been used, giving only a single diastereomeric adduct.

## Conclusion

Our data demonstrate that acylation of the model peptide octreotide occurring in polyester microspheres depends on the hydrophilicity of the polymer and accessibility of the carbonyl ester groups for nucleophile attack by the peptide. The N-terminus of octreotide is most sensitive for acylation, and besides the primary amine of lysine in octreotide also the primary OH of the end group of octreotide was subjected to acylation. In (PC-PEG-PC)-(PL) polymer besides formation of lactic acid adducts, also formation of butanediisocyanate-LA was observed. Understanding the possible sites of acylation can be used to rationally develop methods and/or excipients that inhibit or preferably prevent this unwanted reaction. Several strategies for minimizing and preventing the peptide acylation in PLGA formulations have been proposed and studied in the past, such as the effects of pH-modifying excipients (35), water-soluble divalent cationic salts (17) and PEGylation of the peptide for inhibiting acylation (19). We are also working on inhibition of acylation by protecting the nucleophilic sites of the peptide, which will be the subject of a next publication.

## Electronic Supplementary Material

Below is the link to the electronic supplementary material.

ESM 1 (DOCX 75 kb)

## ABBREVIATIONS

DDT: Dithiothreitol
ESI: Electrospray ionization
GA: Glycolic acid
HMGA: Hydroxymethyl glycolic acid
LA: Lactic acid
LC: Loading capacity
LC-ITMS: Liquid chromatography ion-trap mass spectrometry
LE: Loading efficiency
(PC-PEG-PC)-(PL): Poly(ε-caprolactone)-PEG-poly(ε-caprolactone) - poly(l-lactide) multiblock copolymer
PLGA: Poly(d,l-lactic-*co*-glycolic acid)
PLGHMGA: Poly(d,l-lactic-*co*-glycolic-*co*-hydroxymethyl glycolic acid)
PVA: Polyvinyl alcohol
SEM: Scanning electron microscopy
UPLC: Ultra performance liquid chromatography
