# Supplementary material for: Identification and Assessment of Octreotide Acylation in Polyester Microspheres by LC–MS/MS
Source: Pharm Res. 2015 Apr 2;32(9):3044–54. doi: 10.1007/s11095-015-1685-3 (PMC4526596; doi:10.1007/s11095-015-1685-3)
Supplement: Supplementary file 1 — (DOCX 75 kb) [file 11095_2015_1685_MOESM1_ESM.docx]

**Supporting information**

**Identification and Assessment of Octreotide Acylation in Polyester microspheres by LC–MS/MS**

Mehrnoosh Shirangi^a,c^, Wim E. Hennink^a^, Govert W. Somsen^b^, Cornelus F. van Nostrum^a,*^.

*^a^*Department of Pharmaceutics, Utrecht Institute for Pharmaceutical Sciences, Utrecht University, Utrecht, The Netherlands

*^b^*AIMMS Division of Biomolecular Analysis, VU University Amsterdam, Amsterdam, the Netherlands

^c^ Department of Drug and Food Control, Faculty of Pharmacy, Tehran University of Medical science, Tehran, Iran

*corresponding author: [C.F.vanNostrum@uu.nl](mailto:C.F.vanNostrum@uu.nl)


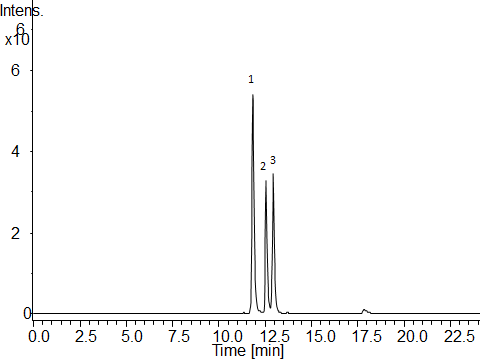


**Fig. S1** Extracted**-**ion chromatogram at m/z 1109 (reduced octreotide +HMGA,1021+88) obtained by LC-MS from octreotide released PLGHMGA after 45 days.


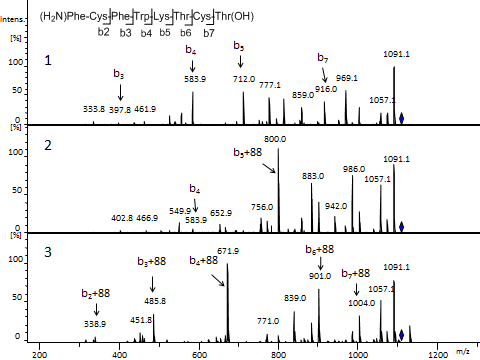


**Fig. S2** MS/MS spectra at m/z 1109 of observed peak in figure S1


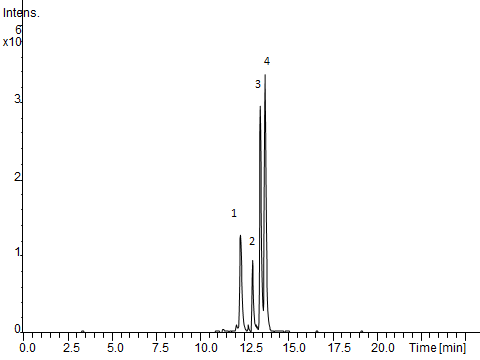


**Fig. S3** Extracted**-**ion chromatogram at m/z 1093 (reduced octreotide +LA,1021+72) obtained by LC-MS from octreotide released PLGHMGA after 45 days.


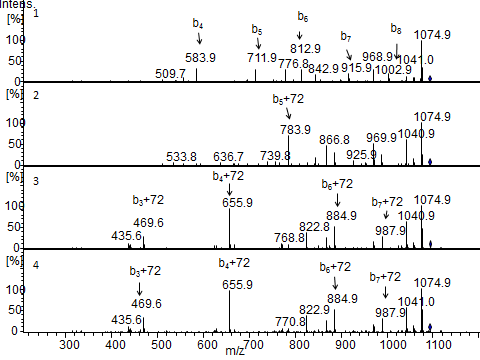


**Fig. S4** MS/MS spectra at m/z 1093 of observed peak in figure S3


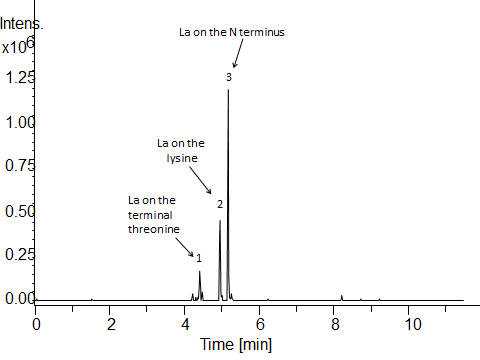


**Fig. S5** Extracted Ion chromatogram at m/z 1091 (octreotide +LA, 1019+72) obtained by UPLC-MS from octreotide released (PC-PEG-PC)-(PL) microspheres
